# Gut-Bone Axis: A Non-Negligible Contributor to Periodontitis

**DOI:** 10.3389/fcimb.2021.752708

**Published:** 2021-11-16

**Authors:** Xiaoyue Jia, Ran Yang, Jiyao Li, Lei Zhao, Xuedong Zhou, Xin Xu

**Affiliations:** ^1^ State Key Laboratory of Oral Diseases, National Clinical Research Center for Oral Diseases, West China Hospital of Stomatology, Sichuan University, Chengdu, China; ^2^ Department of Pediatric Dentistry, West China Hospital of Stomatology, Sichuan University, Chengdu, China; ^3^ Department of Cariology and Endodontics, West China Hospital of Stomatology, Sichuan University, Chengdu, China; ^4^ Department of Periodontology, West China Hospital of Stomatology, Sichuan University, Chengdu, China

**Keywords:** gut microbiota, periodontitis, gut-bone axis, gut epithelial barrier, osteoimmunology, alveolar bone loss

## Abstract

Periodontitis is a polymicrobial infectious disease characterized by alveolar bone loss. Systemic diseases or local infections, such as diabetes, postmenopausal osteoporosis, obesity, and inflammatory bowel disease, promote the development and progression of periodontitis. Accumulating evidences have revealed the pivotal effects of gut microbiota on bone health *via* gut-alveolar-bone axis. Gut pathogens or metabolites may translocate to distant alveolar bone *via* circulation and regulate bone homeostasis. In addition, gut pathogens can induce aberrant gut immune responses and subsequent homing of immunocytes to distant organs, contributing to pathological bone loss. Gut microbial translocation also enhances systemic inflammation and induces trained myelopoiesis in the bone marrow, which potentially aggravates periodontitis. Furthermore, gut microbiota possibly affects bone health *via* regulating the production of hormone or hormone-like substances. In this review, we discussed the links between gut microbiota and periodontitis, with a particular focus on the underlying mechanisms of gut-bone axis by which systemic diseases or local infections contribute to the pathogenesis of periodontitis.

## Introduction

Periodontitis is an irreversible chronic inflammatory disease that typically manifests as the destruction of tooth-supporting tissues, including gingiva, periodontal ligament, and alveolar bone. In severe form, persistent pathologic alveolar bone loss in periodontitis may result in tooth loss and subsequent damage of masticatory function, negatively affecting general health and quality of life. National Health and Nutrition Examination Surveys (NHANES) from 2009 to 2012 reported a high prevalence (up to 46%) of chronic periodontitis in US population aged 30 years and older, among which 8.9% adults suffer from severe periodontitis (Eke et al., 2015). A heavier burden of periodontitis was found in US adults aged 65 years old and above, with 64.1% for mild/moderate periodontitis (Eke et al., 2016). Korea NHANES in 2014 revealed that 41.1% of survey population aged from 40 to 79 years old suffered from periodontitis (Lee and Lee, 2019). Additionally, cross-sectional studies in Norway presented that nearly 50% of population had periodontitis, with 9.1% in severe form or 20.1% in periodontitis stage III/IV (Holde et al., 2017; Bongo et al., 2020). Higher prevalence of severe periodontitis was also found in adult population of North Italy (Aimetti et al., 2015). At present, periodontitis is one of the major public health concerns with a rapidly growing prevalence in middle-aged and elderly population.

Periodontitis is a multifactorial pathological condition mainly induced by oral microbiota. The interactions between host immune responses and periodontal pathogens, such as *Porphyromonas gingivalis*, *Treponema denticola*, and *Tannerella forsythia*, contribute to the pathogenesis of periodontitis (Frederick et al., 2011; Silva et al., 2015). In addition, general health status affects periodontitis. Systemic diseases or local infections of other tissues are critical contributors to periodontal pathology (Genco and Borgnakke, 2013; Reynolds, 2014). As an established risk factor, diabetes mellitus increases the prevalence and severity of periodontitis (Lalla and Papapanou, 2011; Genco and Borgnakke, 2020). Systemic review and meta-analysis showed increased clinical attachment loss in women with postmenopausal osteoporosis (PMO) or osteopenia (ON) compared to healthy women (Penoni et al., 2017). Epidemiological studies showed an increased prevalence of periodontitis and aggravated periodontal bone loss in patients with inflammatory bowel diseases (IBDs), including ulcerative colitis and Crohn’s disease (Papageorgiou et al., 2017; She et al., 2020). Obesity and hyperlipidemia as well as metabolic syndrome are also suggested as risk factors for periodontitis, which increase the risk to gain periodontitis, exacerbate periodontal bone destruction, and are potentially linked with the poor response to non-surgical periodontal therapy (Cavagni et al., 2013; Li et al., 2015; Cavagni et al., 2016; Suvan et al., 2018; Virto et al., 2018). Systemic or intestinal diseases increase the complexity of periodontitis etiology and therapy; however, the underlying mechanisms remain unclear.

## Gut Microbiota and Bone Health

Gut microbiota is the collection of microorganisms residing in the host luminal stream or adhering to the gut mucosa (Zaiss et al., 2019). Regarded as a whole, trillions of gut microorganisms interact with the host *via* releasing various signals, and affect host development, physiology, and general health. Gut microbiota or specific microbial metabolites not only locally influence host inflammatory responses, nutrition intake, or gut barrier function, but also link with host immune system, glucose homeostasis, lipid metabolism, energy balance, non-alcoholic fatty liver disease, adiposity and related comorbidity, and other metabolic diseases (Kamada et al., 2013; Rosenbaum et al., 2015; Morrison and Preston, 2016; Rooks and Garrett, 2016; Li et al., 2017; Fei et al., 2020; Fan and Pedersen, 2021).

### The Role of Gut Microbiota in Bone Turnover

Gut-bone axis refers to the communications between gut microbiota and skeletal system whereby gut microbiota affects bone health. Accumulating evidences have revealed that gut microbiota is a critical factor in bone turnover. The analyses *via* Mendelian randomization approach or polygenetic risk scoring suggest significant association between gut microbiota and human bone mineral density (BMD) in different sites, such as heel or pelvis, by utilizing gut microbiota statistic from genome-wide association study (GWAS) and BMD values from UK biobank cohort (Cheng et al., 2020; Ni et al., 2021). Gnotobiotic animal models directly prove gut microbiota as a key regulator of bone turnover. Germ-free (GF) adult mice exhibit increased both cortical and trabecular bone mass in femur and improved trabecular morphology than conventionally raised controls (Sjögren et al., 2012; Ohlsson et al., 2017). Short-term gut microbial colonization promotes bone turnover and reduces femoral bone mass in GF mice, while adult mice with long-term gut microbial colonization gain enhanced longitudinal and radial growth of femur (Yan et al., 2016). Interestingly, gut microbial colonization induces varied bone phenotypes in GF mice according to the donors of different ages or nutritional status (Blanton et al., 2016), possibly resulting from the variance of gut microbial composition. Emerging evidences have indicated the impact of specific gut microbe segmented filamentous bacteria (SFB) on bone growth and maturation (Hathaway-Schrader et al., 2020; Tyagi et al., 2021). Compared to GF mice, SFB-monoassociation caused reduced bone loss and inferior trabecular morphology in the tibia of mice (Hathaway-Schrader et al., 2020). Similarly, a complex gut microbiota colonization within SFB also induced an osteopenic tibial phenotype in mice compared to gut microbiota colonization devoid of SFB (Hathaway-Schrader et al., 2020). The negative effects of SFB on skeletal maturation have been also proved *via* cohabitation, fecal material transplantation, and maternal/offspring transmission models, further indicating gut microbiota as a non-genomic hereditary factor in shaping offspring bone phenotype (Tyagi et al., 2021). In addition, the disruption of gut microbial homeostasis due to antibiotic intervention alters bone mass and biomechanical properties, further suggesting the regulation of gut microbiota on physiological bone remodeling (Yan et al., 2016; Rios-Arce et al., 2020).

### The Role of Gut Microbiota in Bone diseases

#### Osteoporosis/Osteopenia

Osteoporosis (OP) is a systemic skeletal disease characterized by decreased bone mass and microarchitecture destruction, with elevated risk of fracture (Glaser and Kaplan, 1997). PMO induced by estrogen deficiency is the most common clinical form, and OP can be also secondary to medication or other systemic diseases, such as hypercortisolism and hyper-parathyroidism (Glaser and Kaplan, 1997). Osteopenia (ON) is an abnormal state with low bone density but not as severe as osteoporosis (Karaguzel and Holick, 2010). Estrogen deficiency due to gonadotropin-releasing hormone agonists induced no significant bone loss in GF mice, suggesting gut microbiota as a key determinant in PMO development (Li J.Y et al., 2016). PMO or ON patients showed distinct alterations in gut microbial taxa and metabolites compared to healthy individuals, including elevated levels of phylum *Gemmatimonadetes* and *Chloroflexi*, as well as genera *Blautia*, *Parabacteroides*, and *Ruminococcaceae* (Wang et al., 2017; Das et al., 2019; He et al., 2020). In addition, the genus *Bacteroides* and family *Rikenellaceae* were linked with high risk of fracture and low BMD in Japanese postmenopausal women (Ozaki et al., 2021). Glucocorticoid (GC) caused bone loss along with altered gut microbiota in mice, which can be prevented by antibiotic or probiotic treatment (Schepper et al., 2020). The mice that received fecal material from GC-treated subjects also showed pathological bone loss, supporting the involvement of gut microbiota in GC-induced osteoporosis (Schepper et al., 2020). In addition, the gut taxa SFB-dependent gut-bone crosstalk is also involved in the hyperparathyroidism-induced bone loss (Yu et al., 2020).

#### Osteoarthritis

Osteoarthritis (OA) is the most common degenerative joint disease. The results in both Rotterdam Study and LifeLines-DEEP cohort showed an association between higher abundance of gut *Streptococcus* spp. and increased OA-related knee pain and joint inflammation (Boer et al., 2019). Another pilot study also demonstrated a distinct alteration in gut microbiota composition in knee OA patients compared to healthy individuals (Ramasamy et al., 2021). Additionally, animal models demonstrated the involvement of gut microbiota in OA development as well as the association between OA severity and certain gut genera, including *Fusobacterium*, *Faecalibacterium*, and *Ruminococcacea* (Huang et al., 2020).

### The Involvement of Gut Microbiota in Periodontitis

The studies by Uchida and Irie reported that commensal gut microbiota-colonized mice showed increased alveolar bone loss and compromised trabecular morphology compared with GF mice (Irie et al., 2018; Uchida et al., 2018). Compared to the rats with normal diet, obese-insulin-resistant rats fed with high-fat diet presented with enhanced osteoclast-mediated bone resorption, decreased jawbone mineral density, as well as impaired jawbone microarchitecture, and the jawbone morphology could be improved by the administration of probiotics, prebiotics, or symbiotics supplements (Eaimworawuthikul et al., 2019; Eaimworawuthikul et al., 2020) (**Table 1**).

**Table 1:** The effects of gut microbiota on alveolar bone physiology and periodontitis.

Alveolar bone physiology/periodontitis	Association between gut microbiota and bone	
Alveolar bone physiology	GF mice, alveolar bone mass↑, trabecular morphology↑ (*vs* SPF mice)	Irie et al., 2018; Uchida et al., 2018
Alveolar bone physiology	Probiotics/prebiotics/symbiotics gavage, obese-insulin resistance-induced alveolar bone loss↓	Eaimworawuthikul et al., 2019, 2020
Periodontitis	*P. gingivalis* gavage, periodontitis severity↑;	Palioto et al., 2019; Mulhall et al., 2020
	*P. gingivalis* gavage in combination with *A. muciniphila* and its pili-like protein, periodontitis severity↓	
Obesity-related periodontitis	Obese mice, periodontitis severity↑;	Huck et al., 2020
	*A. muciniphila* gavage, obesity-related periodontitis↓	
PMO-related periodontitis	PMO rats, butyrate-producing bacteria↓, periodontitis severity↑;	Jia et al., 2019, 2021
	PMO rats with berberine or probiotics gavage, butyrate-producing bacteria↑, periodontitis severity↓	

PMO, postmenopausal osteoporosis; GF, germ-free; SPF, special pathogen free.

Periodontitis is a complex inflammatory condition in periodontium with multiple contributory factors. Although periodontal microbiota drives the onset of periodontitis, systemic diseases or local infections of other tissues, such as PMO, obesity, diabetes mellitus, and IBDs, can promote the progression of periodontal destruction. Gut microbiota potentially provides critical links between periodontitis and general health. The mice treated by *P. gingivalis* gavage alone or in combination with local ligation exhibited increased alveolar bone loss than those with simply ligation-induced periodontitis, and gut commensal microbe *Akkermansia muciniphila* or its pili-like protein Amuc_1100 protected the mice gavaged with *P. gingivalis* from alveolar bone loss in periodontitis (Palioto et al., 2019; Mulhall et al., 2020). The protective role of *A. muciniphila* on alveolar bone was also observed in obese murine model (Huck et al., 2020). Obese mice showed elevated alveolar bone loss in *P. gingivalis*–induced periodontitis than lean mice, and gut commensal microbe *A. muciniphila* supplement by oral gavage significantly reduced obese-related alveolar bone loss in periodontitis (Huck et al., 2020). Our previous studies reported that estrogen deficiency due to ovariectomy (OVX) led to decreased abundance of gut butyrate-producing bacteria as well as enhanced periodontal bone loss in periodontitis rats, and that berberine or probiotics supplements enriched specific gut butyrate-producing genera and prevented OVX-induced alveolar bone resorption in periodontitis, further suggesting that PMO can affect periodontitis *via* regulating gut microbiota (Jia et al., 2019; Jia et al., 2021) (**Table 1**).

## Underlying Mechanisms of Gut-Alveolar Bone Axis

Bidirectional regulations between gut and oral cavity account for the complex pathologies and interaction of gut and oral diseases. The translocation of oral pathogens to gut *via* alimentary canal or hematogenous way contributes to gut physiology or pathology (Kitamoto et al., 2020a) . The patients suffering from gut diseases, including irritable bowel syndrome, IBD, and colorectal cancer, showed an enrichment of oral pathobionts, such as the family *Enterobacteriaceae*, *Peptostreptococcaceae*, *Pasteurellaceae*, and *Veillonellaceae*, as well as the genus *Klebsiella*, *Porphyromonas*, *Fusobacterium*, and *Streptococcus* (Geng et al., 2014; Gevers et al., 2014; Pittayanon et al., 2019; Kitamoto et al., 2020a). The mice with periodontitis exhibited ectopic gut colonization of oral pathobionts belonging to *Enterobacteriaceae*, which could induce TH1 immune responses and directly aggravate the gut inflammation in colitis (Atarashi et al., 2017; Kitamoto et al., 2020b). Additionally, periodontitis indirectly exacerbates gut inflammation *via* the gut homing of oral pathobiont-reactive Th17 cells (Kitamoto et al., 2020b). The crosstalk between oral microbiota and gut diseases in “oral-gut” axis explicitly indicates the effect of oral inflammation on gut health. However, the potential mechanisms whereby gut microbiota regulates oral health in “gut-oral” axis remain uncertain. Mounting evidences provide multiple mechanisms potentially involved in “gut-alveolar bone” axis, supporting the role of gut microbiota in periodontitis development.

### Microbial Pathway

**Figure 1:**
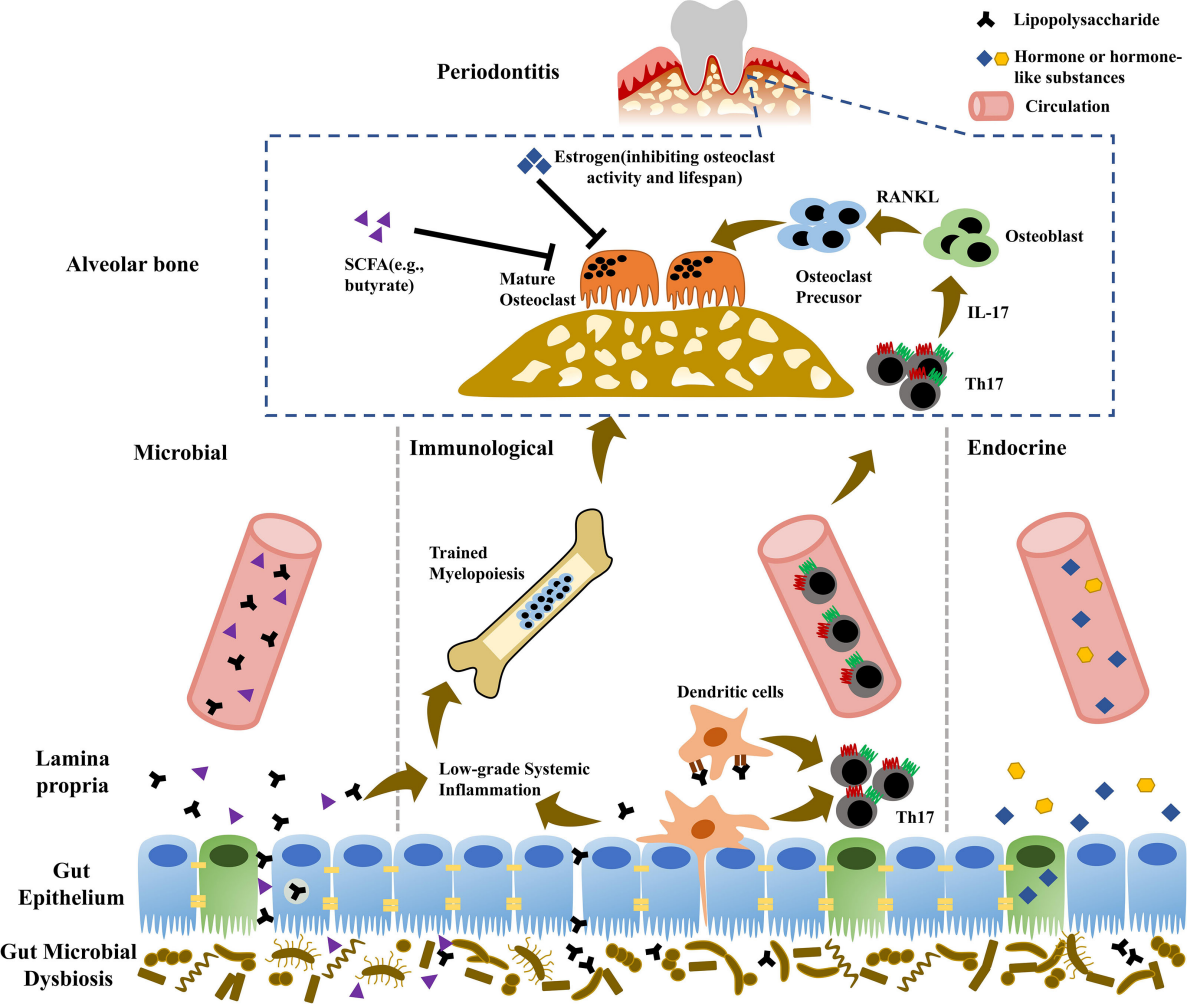
Potential mechanisms involved in “gut-alveolar bone” axis. Microbial pathway: due to impaired gut barrier, gut pathobionts or metabolites possibly translocate to distant alveolar bone *via* hematogenous way, provoking local inflammatory responses and aggravating periodontitis. Immunological pathway: gut pathogens can induce the expansion of gut pathogenic Th17 cells, which potentially migrate to alveolar bone and promote periodontitis development. Additionally, elevated systemic inflammation burden due to microbial translocation enhances trained myelopoiesis in the bone marrow with increased generation of neutrophils and monocyte lineage, which are recruited to periodontal tissue and exacerbate periodontitis. Endocrine pathway: gut microbiota can regulate the production of human hormone or hormone-like chemicals (e.g., growth hormone, insulin-like growth factors, and gonadal steroids), which further influence bone homeostasis and periodontitis.

Ectopic gut colonization of oral pathogens mainly accounts for the effects of oral inflammation on gut health. Similarly, gut pathobionts and metabolites potentially translocate to distant organs in hematogenous way and directly affect general health. The translocation of gut microbes and metabolites across gut epithelium includes transcellular and paracellular ways. Enterocyte phagocytosis is responsible for transcellular translocation. Paracellular pathway depends on the permeability of gut epithelium, the barrier physically resisting exogenous pathogens and selectively allowing the passage of nutrients. Tight junction (TJ) underlies the paracellular pathway of gut epithelial barrier, which consists of TJ protein complexes including claudin, occludin, and zonula occludens (ZO) proteins. Multiple pathologies can impair gut epithelial barrier function *via* modulating the production and distribution of TJ proteins, eliciting the “leakage” of gut pathobionts or metabolites into systemic circulation and microbial expansion to distant organs (**Figure 1**).

#### Lipopolysaccharide

Lipopolysaccharides (LPS), the important component of lipoproteins derived from Gram-negative gut microbes, is a strong stimulatory endotoxin triggering inflammatory immune responses and affecting general health. As a result of enrichment of gut LPS-containing microbes and increased gut paracellular permeability, gut-derived LPS expansion in circulation, defined as endotoxemia, or LPS translocation in distant tissues can potentially induce low-grade inflammation and promote the development of systemic disorders, such as Parkinson’s disease, obesity, insulin resistance, diabetes, NAFLD, ST-elevation myocardial infarction, and atherosclerosis (Cani et al., 2007; Cani et al., 2008; Geng et al., 2016; Perez-Pardo et al., 2019; Carnevale et al., 2020; Cho et al., 2021). In addition, GF mice model further provided strong evidence for the causative role of LPS in NAFLD development, as indicated by that only simultaneous high-fat diet administration and endotoxin-producing pathobiont mono-association induce NAFLD (Fei et al., 2020). The administration of gut commensal microbes, such as *A. muciniphila* and *Roseburia intestinalis*, and the modulation of gut microbiota *via* certain natural substances induced improved gut barrier function and attenuated LPS-associated systemic inflammation, consequently improving related systemic diseases (Cani et al., 2009; Jiang et al., 2016; Li J. et al., 2016; Song et al., 2016; Kasahara et al., 2018; Bian et al., 2020). In addition to paracellular pathway, LPS can translocate gut barrier in transcellular way *via* the phagocytosis of enterocyte mediated by toll-like receptor 4 (TLR4) (Neal et al., 2006). The obese rats administrated by natural pectin exhibited both increased TJ protein expression and reduced TLR4 expression in the ileum, leading to attenuated systemic inflammation (Jiang et al., 2016).

In gut-bone axis, leaky gut-induced LPS translocation and systemic inflammation potentially link to bone pathologies. Transplantation of fecal materials from metabolic syndrome patients in GF mice enhanced gut permeability by downregulating TJ proteins ZO-1 and occludin, causing elevated plasma level of LPS and subsequently exacerbated OA severity (Huang et al., 2020). Antibiotic treatment altered gut microbiota and decreased serum LPS level, alleviating circulating inflammation and preventing OA progression (Guan et al., 2020). Estrogen-deficient mice exhibited pathological bone loss and impaired bone microarchitecture in femur, with downregulated gut epithelial TJ protein expression and increased serum endotoxin levels, and these pathologies could be prevented by probiotics administration (Li J. Y. et al., 2016). Consistently, our previous studies observed enhanced gut permeability, elevated serum LPS, and systemic inflammation in OVX mice, consequently resulting in Th17-related immune responses in alveolar bone and aggravated periodontitis (Jia et al., 2019; Jia et al., 2021). In addition, administration of probiotics or berberine increased gut butyrate-producing bacteria, enhanced gut barrier function with reduced circulating LPS, and thus ameliorated estrogen deficiency-induced alveolar bone loss (Jia et al., 2019; Jia et al., 2021). Estrogen is potentially an important modulator of gut barrier. 17β-estradiol (E2), the predominant form of estrogen, can directly enhance gut barrier function by upregulating the expression of gel-forming mucin 2 (MUC2) and TJ proteins (ZO-1, occludin, and claudin 4) in colonic mucosa upon estrogen β signaling (Song et al., 2018). Additionally, estrogen can indirectly affect gut barrier by modulating gut microbiota. Estrogen deficiency induces reduction in gut butyrate-producing bacteria (Jia et al., 2019; Jia et al., 2021). Butyrate is a beneficial bacterial metabolite that reinforces gut barrier (Geirnaert et al., 2017). As a result, increased gut permeability due to estrogen deficiency induces gut pathogens translocation into circulation, causing inflammatory responses and promoting alveolar bone loss. Thus, gut barrier can be a potential therapeutic target for periodontitis under estrogen deficiency. Probiotics, such as *Lactobacilli* and *Bifidobacteria*, are live bacteria that benefit the host if provided as dietary or medical supplements in adequate quantities. Berberine is an alkaloid present in some medicinal plants, such as *Berberis vulgaris*, and *Hydrastis canadens*. With selective enrichment of gut butyrate-producing bacteria, probiotics and berberine supplements enhance gut barrier function and exert protective roles on alveolar bone under estrogen deficiency, representing a promising adjuvant treatment against periodontitis in postmenopausal women.

#### Short-Chain Fatty Acids

Short-chain fatty acids (SCFAs) are end-products deriving from gut microbial fermentation of dietary indigestible fibers, which escape from host digestion and absorption, mainly including butyrate, propionate, and acetate (Koh et al., 2016; Morrison and Preston, 2016). The type and amount of SCFAs generated in the gut depend on gut microbial composition, substrate types, and intestinal transit time (Macfarlane and Macfarlane, 2003). SCFA concentration varies among different segments of human intestinal tract, with high level in the cecum and proximal colon (Cummings et al., 1987). SCFAs are absorbed in the cecum and colon *via* active transport mediated by Na^+^- and H^+^-coupled monocarboxylate transporters, or *via* protonation-dependent non-ionic diffusion, or possibly *via* special exchange with intracellular bicarbonate or other SCFAs (Titus and Ahearn, 1988; Canfora et al., 2015; Koh et al., 2016). In addition to being partially metabolized by colonocytes as energy source, other absorbed SCFAs translocate into circulation. Although distinctly lower than the concentration in colonic contents, three major SCFAs are detected in human portal, hepatic, and peripheral venous blood with a descending trend among the levels of acetate, propionate, and butyrate (Cummings et al., 1987). Consistently, propionic and butyric acid levels in cecal contents are also correlated with that in aortic serum of the rats fed with dietary fibers (Jakobsdottir et al., 2013).

*Via* hematogenous way, gut-derived SCFAs are potentially transmitted to distant organs and involved in physiological processes or diseases. Transported through portal circulation to liver, SCFAs are consumed by hepatocytes in energy metabolism as well as the biosynthesis of cholesterol, glucose, and fatty acids (Dalile et al., 2019). Following hepatic utilization, small amounts of SCFAs travel to other organs *via* systemic circulation. SCFAs translocate to brain across blood-brain barrier and affect brain physiology and pathology, such as central appetite regulation, and post-stroke improvement (Frost et al., 2014; Lee et al., 2020). SCFAs generated by maternal gut microbiota are reflected in embryo tissues or organs, including sympathetic nerves, gut epithelium, and pancreas, participating in the development of postnatal nerve and metabolic systems *via* GPR41 and GPR43 signaling (Kimura et al., 2020).

Gut-derived SCFAs, especially butyrate and propionate, are the regulators of bone homeostasis, traveling to bone marrow and directly affecting bone metabolism. Fructo-oligosaccharide administration significantly increases serum level of butyrate and promotes bone formation in rats, ameliorating the pathological bone loss due to estrogen deficiency (Porwal et al., 2020). Study by Lucas and colleagues directly demonstrated the translocation of SCFAs to bone tissue, as evidenced by augmented butyrate or propionate concentrations in bone marrow of the mice with exogenous supplement of corresponding SCFA in drinking water (Lucas et al., 2018). Previous studies proved the direct regulation of SCFAs on the differentiation of osteoclasts and osteoblasts (Iwami and Moriyama, 1993; Rahman et al., 2003; Chen et al., 2007; Morozumi, 2011; Chang et al., 2016; Lucas et al., 2018). Butyrate, the strong histone deacetylase inhibitor, have been reported as a strong inhibitor of osteoclastogenesis. Early study showed the inhibitory role of sodium butyrate on murine osteoclast differentiation in bone marrow, possibly depending on the cytotoxicity of sodium butyrate (Iwami and Moriyama, 1993). Subsequent researches further updated related specific mechanisms. The *in vitro* study on murine macrophage cell line RAW264 showed that sodium butyrate inhibited osteoclast formation *via* regulating nuclear factor-κB (NF-κB) and mitogen-activated protein kinase (MAPK) signaling pathways (Rahman et al., 2003). Furthermore, butyrate and propionate can inhibit osteoclast differentiation *via* enhancing glycolysis in osteoclast precursors and subsequently downregulating the expression of osteoclast marker genes TRAF6 and NFATc1 (Lucas et al., 2018). The role of SCFAs on osteoblast formation remains uncertain. Sodium butyrate can promote osteogenic differentiation of mesenchymal stem cells *via* activating ERK pathway (Chen et al., 2007). However, osteoblastic differentiation of ROS17/2.8 cells was halted in the presence of high-level butyrate (10^-3^M) (Morozumi, 2011). MG-63 osteoblasts treated by butyrate demonstrated suppressed cell proliferation with increased cell cycle arrest and reactive oxygen species (ROS) production (Chang et al., 2016). However, the *in vivo* study by Lucas showed no effects of SCFAs on osteoblasts and bone formation (Lucas et al., 2018).

Our previous studies indicated that estrogen deficiency led to reduction of gut butyrate-producing bacteria, such as *Clostridium leptum*, *Clostridium coccoides*, *Fecalibacterium prausnitzii*, and *Roseburia*, as well as decreased butyrate production, leading to more severe periodontitis (Jia et al., 2019; Jia et al., 2021). Rescue of gut butyrate-producing bacteria and butyrate production by berberine or probiotics prevented the alveolar bone loss induced by estrogen deficiency, suggesting the pivotal roles of gut-derived butyrate in periodontitis (Jia et al., 2019; Jia et al., 2021). The indirect regulation of gut-derived butyrate on periodontitis possibly depends on gut barrier. In addition, butyrate may potentially translocate to alveolar bone *via* circulation and directly affects periodontitis progression.

Of note, some pathogenic bacteria in periodontitis, such as *Porphyromonas gingivalis* and *Fusobacterium nucleatum*, can produce SCFAs, including butyrate and propionate. Interestingly, butyrate has cytotoxic effects in patients with periodontitis. The concentration of butyrate in gingival crevices positively correlates to the severity of periodontitis. Butyric acid could reach to millimolar concentration in the gingival crevicular fluid of periodontitis patients, whereas the maximum plasma concentration of butyrate of adult leukemia patients after intravenous infusion only reached to 0.05 millimolar (Miller et al., 1987; Niederman et al., 1997; Lu et al., 2014). Oral-derived butyrate can promote periodontitis development (Guan et al., 2021), likely due to its cytotoxic effects on gingival epithelial cells and fibroblasts. Butyrate at millimolar concentrations can induce apoptosis, autophagy, and pyroptosis of gingival epithelial cells and impair epithelial TJ, leading to the destruction of gingival epithelial barrier and periodontitis initiation (Tsuda et al., 2010; Evans et al., 2017; Liu et al., 2019; Magrin et al., 2020). Butyric acid can negatively affect cell growth and cell cycle progression of gingival fibroblasts by inducing reactive oxygen species production (Kurita-Ochiai et al., 2008; Chang et al., 2013), and lead to mitochondria- and caspase-dependent apoptosis in inflamed gingival fibroblasts in a dose-dependent manner (Kurita-Ochiai et al., 2008). Long-term exposure to butyrate can also lead to cytostasis and apoptosis of healthy gingival fibroblasts *via* intrinsic and extrinsic pathways, promoting periodontitis progression (Shirasugi et al., 2017; Shirasugi et al., 2018). In addition, butyrate can dose-dependently induce the production of proinflammatory cytokines in gingival fibroblasts, including IL-6 and IL-11, which rescue T-cell from apoptosis and contributed to the development of periodontitis (Kurita-Ochiai et al., 2002). Thus, unlike circulating butyrate derived from gut microbiota, local accumulation of concentrated butyrate due to periodontal pathogens has deleterious effects on alveolar bone, which should be taken into consideration in future investigation.

### Immunological Pathway

#### The Immune Responses Involved in Periodontitis

The bone homeostasis is the dynamic balance between the coupled processes of osteoblast-mediated bone formation and osteoclast-mediated bone resorption. The immune system has long been recognized as an essential regulator of bone metabolism. The crosstalk between immune and skeletal systems, termed as “osteoimmunology,” underlies bone physiological and pathological processes (Rho et al., 2004). The components of immune systems, such as immune cells and cytokines, are involved in bone remodeling and metabolic or inflammatory bone diseases, including osteoporosis, arthritis, and periodontitis (Weitzmann and Pacifici, 2006; Hajishengallis, 2015; Kuhn and Morrison, 2020). Periodontitis is an inflammation induced by exogenous pathogens. Microbial pathogens are recognized and presented by innate immune system to activate adaptive immune responses, thereby inducing the production of effector cells and molecules and driving the initiation of periodontal destruction.

##### Neutrophil

Neutrophils are terminally differentiated white blood cells produced abundantly in the bone marrow and recruited to infected or damaged tissues *via* circulation (Borregaard, 2010). Neutrophils are functionally versatile effector cells, displaying antibacterial and cytotoxic properties, as well as *de novo* biosynthesis of chemokines and cytokines with multiple functions (e.g., proinflammatory, anti-inflammatory, and immunoregulatory) (Scapini and Cassatella, 2014). Apart from acute infections, it has been currently well established that neutrophils are also implicated in chronic inflammatory diseases such as periodontitis (Hajishengallis, 2020). Neutrophils are integral to maintain periodontal homeostasis *via* transmigrating into gingival crevice and constituting the first defense line against subgingival bacterial plaque (Hajishengallis and Hajishengallis, 2014). The neutrophils with hyperactivity or in excessive amount contribute to periodontal tissue destruction and periodontitis progression (Hajishengallis et al., 2015). In addition, neutrophils are capable of producing chemokines CCL2 and CCL20, selectively recruiting pathogenic T-helper 17 (Th17), the pivotal cells in periodontitis development (Pelletier et al., 2010).

##### T-Helper 1 Cell and T-Helper 2 Cell

T-helper 1 cell (Th1) and Th2 are the major effector cells responsible for cellular immunity and humoral immunity, respectively. Th1/Th2 paradigm mainly accounts for the mechanism of periodontitis progression. Th1 cells are predominant in gingivitis or stable periodontal lesion, while established or progressive periodontal lesion in chronic periodontitis is mediated by Th2 cells, which subsequently activate B cell/plasma cell response (Ohlrich et al., 2009). T and B lymphocytes highly express receptor activator for NF-κB ligand (RANKL) and promote RANKL-mediated osteoclast differentiation in periodontitis (Kawai et al., 2006).

##### Th17 and Regulatory T Cell

Accumulating evidences have indicated the pivotal roles of novel T subsets Th17 and regulatory T cell (Treg) in the pathogenesis of bone diseases, such as osteoporosis, autoimmune arthritis, and ankylosing spondylitis (Komatsu et al., 2014; Li J. Y. et al., 2016; Schmidt et al., 2019; Xu et al., 2019; Xie et al., 2020). Th17 and Treg cells share common precursor cells and cytokines for initial differentiation, but result in opposite functions. Th17 cells are the T-cell subset producing IL-17 and triggering immune responses against exogeneous pathogens, while Treg cells suppress immune responses for immune homeostasis. In consistent with Th1/Th2 cells balance, Th17/Treg cells are maintained in dynamic equilibrium, while Th17/Treg shifting in favor of Th17 elicits pathological conditions. Th17/Treg imbalance is involved in periodontitis development, as indicated by enhanced Th17 response with increased IL-17A production and suppressed anti-osteoclastogenic function of Treg, which is also associated with exacerbated periodontitis during pregnancy (Hays et al., 2019; Alvarez et al., 2020). Additionally, *Aggregatibacter actinomycetemcomitans*–induced periodontitis mice show strong Th17 immune responses in periodontal lesions (Monasterio et al., 2018). The orthodontic tooth movement under periodontitis depends on osteoclast metabolism modulated by Th17/Treg equilibrium (Ge et al., 2020). In addition, Th17 proportion in periodontal lesions from the patients with periodontitis is distinctly higher than that in healthy individuals and correlated with the severity of periodontitis, while Th17 cell defects reduce susceptibility to periodontitis (Dutzan et al., 2018). Oral pathogens drive pathogenic Th17 differentiation in periodontitis *via* interleukin-6 (IL-6) and IL-23 signaling (Dutzan et al., 2018; Bunte and Beikler, 2019). Pathogenic Th17 cells further induce mucosal immune response and periodontal bone destruction to eradicate pathogens at the expense of bone damage (Tsukasaki et al., 2018). Our previous studies explored Th17/Treg imbalance in the pathogenesis of periodontitis under estrogen deficiency (Jia et al., 2019; Jia et al., 2021). Estrogen-deficient rats demonstrated exacerbated bone loss in periodontitis with increased Th17 cells or an elevated Th17/Treg ratio in both alveolar bone and femoral bone marrow, while probiotics or berberine administration rescued Th17/Treg imbalance and prevented estrogen deficiency-induced alveolar bone destruction in periodontitis (Jia et al., 2019; Jia et al., 2021). In addition, IL-17 derived from Th17 can amplify the production of RANKL by osteoblasts, indirectly inducing osteoclast differentiation (Hajishengallis and Chavakis, 2021).

#### Potential Pathways in the “Gut-Immunity-Periodontitis” Axis

##### Gut-Derived Lymphocytes Migration

Multiple lymphocytes are evoked in intestinal lamina propria and migrate to effector sites depending on the mediation of chemokines, participating in the development, physiology, and pathogenesis of distant tissues (Gray et al., 2017; Huang et al., 2018). In addition to microbial translocation, gut-derived immunocytes migration to bone tissue is an alternative pathway in “gut-bone” axis. Th17 cells abundantly reside in intestinal lamina propria. Specific gut microbes induce the differentiation of Th17 cells with distinct functions, namely, homeostatic tissue-resident type and pathogenic inflammatory type (Omenetti et al., 2019). Homeostatic Th17 cells with non-pathogenic plasticity are elicited by gut commensal segmented filamentous bacteria and function as quiescent or memory T cells, maintaining gut barrier integrity and homeostasis (Stockinger and Omenetti, 2017; Ladinsky et al., 2019; Omenetti et al., 2019). During the infection of intestine, gut pathogens, such as *Citrobacter rodentium*, induce intestinal epithelial cells to generate ROS, which promotes pathogenic Th17 differentiation and impairs gut barrier, as followed by translocation of pathogens to the lamina propria and further enhancement of inflammatory Th17 response (Stockinger and Omenetti, 2017). Gut pathogenic Th17 cells migrate to extraintestinal tissues *via* circulation, contributing to the development of multiple diseases. The migration of Th17 can be directly proved using transgenic mice expressing photoconvertible fluorescence protein Kaede, an ideal system to track lymphocyte movement (Morton et al., 2014; Krebs et al., 2016; Calcinotto et al., 2018; Mathies et al., 2018). Kaede is fluorescent green and can be photoconverted to red by violet or UV light exposure (Tomura et al., 2008). Accordingly, the cells in the region of interest can be labeled by photoconverted Kaede protein *in vivo* (Tomura et al., 2008). Intestine infection with *C. rodentium* can provoke the expansion of gut pathogenic Th17 cells, which migrate into the kidney and aggravate the pathology in experimental crescentic glomerulonephritis mice (Krebs et al., 2016). During T cell-mediated colitis, pathogenic Th17 migration and absence of Treg are responsible for colitis-associated liver inflammation (Mathies et al., 2018). Gut-derived inflammatory Th17 cells migrate to spleen and interact with B cells, promoting autoantibody production and inducing autoinflammatory arthritis (Morton et al., 2014). Gut pathogenic Th17 cells elicited by *Prevotella heparinolytica* migrate to bone marrow and accelerate multiple myeloma progression in cooperation with eosinophils (Calcinotto et al., 2018). Additionally, gut Th17 migration into the lung also contributes to the development of pulmonary complications during arthritis (Bradley et al., 2017). Furthermore, parathyroid hormone (PTH)-induced bone loss depends on gut microbe SFB capable of inducing Th17 cells (Yu et al., 2020). With the enrichment of gut SFB, PTH promotes the expansion of gut TNF^+^ T cells and Th17 cells, which are recruited from gut to bone marrow and contribute to bone loss (Yu et al., 2020). Although there still lacks direct evidence, gut Th17 migration to alveolar bone is possible, thus potentially contributing to the development of periodontitis (**Figure 1**).

Gut-derived immunocytes migration to distant tissues contains the translocation from intestinal lamina propria to circulation and following migration from circulation to effector tissue. Sphingosine-1-phosphate (S1P) is the major regulator of lymphocyte migration out of lymph nodes. Depending on gradient concentration of S1P, effector T cells expressing S1P receptor-1 (S1PR1) emigrate from lymph node to circulation (Benechet et al., 2016; Yu et al., 2021). In addition, T cells further migrate to distant tissues *via* expressing various chemokine receptors, including CCR subfamily (e.g., CCR4, CCR5, CCR6, CCR7) and CXCR subfamily (e.g., CXCR3, CXCR5, CXCR6), which interacting with the corresponding chemokines (e.g., CCL22, CCL20, CCL21, CXCL9, CXCL10) (Sokol and Luster, 2015). CCR6 signaling regulates the egress of Treg and Th17 to kidney, involving in the development of glomerulonephritides (Turner et al., 2010). Intestinal pathogenic Th17 cells can emigrate out of intestinal lamina propria *via* S1P/S1PR1 signaling, and then travel to the kidney guided by CCL20/CCR6 axis, promoting the pathology of glomerulonephritides (Krebs et al., 2016). Additionally, the influx of gut TNF^+^ cells and Th17 cells to bone marrow induced by PTH or estrogen deficiency is respectively mediated by CXCR3 and CCL20 signaling (Yu et al., 2020; Yu et al., 2021).

##### Trained Myelopoiesis in the Bone Marrow

Estrogen-deficiency-associated low-grade systemic inflammation, possibly induced by impaired gut barrier and hematogenous dissemination of gut pathogens or metabolites, contributes to development of bone pathologies including periodontitis (Li J.Y. et al., 2016; Jia et al., 2019; Jia et al., 2021). However, the underlying mechanism whereby elevated systemic inflammation burden destroys bone homeostasis remains unclear. Bone marrow, the primary site of hematopoiesis, can quickly sense and respond to systemic inflammation, eliciting a cascade of reactions (Hajishengallis and Chavakis, 2021). Historically, innate and adaptive immunity are classic immune forms resistant to infection or injury. In addition to innate and adaptive immunity, trained immunity is a novel immune mode induced by microorganisms or inflammation. Trained immunity could “train” innate immune cells to produce highly reactive and non-specific immune memory *via* epigenetic modifications and metabolic reprogramming, eliminating pathogens and resisting secondary infection (Netea et al., 2016; Netea et al., 2020). Non-innate immune cells, such as hematopoietic stem and progenitor cells (HSPCs), could be also involved in training immunity. Trained immunity initiated in the bone marrow can enhance myelopoiesis with expansion and myeloid-biased differentiation of HSPCs, increasing the production of effector cells against infection, such as neutrophils, and monocytes (Mitroulis et al., 2018; Chavakis et al., 2019). Intravenous Bacillus Calmette-Guérin (BCG) in mice can induce the proliferation and differentiation of hematopoietic stem cells (HSCs) and multipotent progenitors in bone marrow into monocyte/macrophage lineage, resisting the infection of *Mycobacterium tuberculosis* (Kaufmann et al., 2018). In addition to resisting infection and promoting host survival, trained immunity potentially has a harmful impact on the host. Neutrophils or monocytes/macrophages generated in training immunity can be recruited to other infection or injury sites to promote chronic inflammation and the development of systemic diseases such as obesity and atherosclerosis (Bekkering et al., 2020; Schloss et al., 2020). Monocyte lineage, the osteoclast precursors, may migrate to the sites of active bone resorption (e.g., alveolar bone in periodontitis) and differentiate into osteoclasts *via* macrophage colony-stimulating factor (M-CSF) and RANKL signaling, further aggravating periodontal bone loss (Hajishengallis and Chavakis, 2021). Similarly, excessive amounts of neutrophils are possibly recruited to periodontal tissue and exacerbate periodontitis. Of note, periodontitis itself can also induce low-grade systemic inflammation due to hematogenous dissemination of periodontal pathogens or the expansion of periodontal inflammatory mediators (Hajishengallis and Chavakis, 2021). It suggests that periodontitis-associated systemic inflammation potentially in turn aggravated periodontal bone destruction (**Figure 1**).

### Endocrine Pathway

Currently, hormone or hormone-like chemicals, such as growth hormone, PTH, insulin-like growth factors (IGFs), and gonadal steroids, have been considered as important regulators of bone metabolism. Gut microbiota, newly referred as an “endocrine organ”, regulates the production of human hormone and hormone-like substances, influencing host physiology and disorders (**Figure 1**).

#### Insulin-Like Growth Factor-1

IGF-1 belongs to the IGF family, which plays an important role in childhood growth and has anabolic effects in adults. IGF-1 in serum, existing as a complex containing IGF-1 molecule, IGF binding protein 3 (IGFBP-3), and acid labile subunit (ALS), is mainly generated by hepatocytes in response to growth hormone and travels to distant organs *via* circulation (Daughaday et al., 1972). In addition, IGF-1 is also locally produced *via* autocrine or paracrine fashion in non-hepatic organs/tissues, including bone. Accumulating evidences have suggested IGF-1 as an important regulator of skeletal development and bone remolding (Yakar et al., 2010; Tahimic et al., 2013; Wang et al., 2013). With the absence of ALS, reduction of circulating IGF-1 results in disrupted linear growth and decreased bone mineral density (Yakar et al., 2002). Circulating IGF-1 also affects skeletal integrity and mechanical properties *via* regulating lateral growth of long bone and deposition of mineralized cortical bone tissue (Yakar et al., 2009; Courtland et al., 2011). Subcutaneous administration of IGF-1 improves bone density and cortical thickness in osteopenia mice by upregulating osteogenic-related proteins and reducing protein expression in osteoclastic activity (Guerra-Menéndez et al., 2013). Of note, reduced circulating IGF-1 during early lifetime can be compensated by local IGF-1 signaling, as evidenced by increased bone mass with early-life loss of circulating IGF-1 in female mice (Ashpole et al., 2016). IGF-1 binds to IGF-1 receptor (a tyrosine kinase receptor) and elicits IGF-1 signaling, which regulates the growth and differentiation of osteoclasts and osteoblasts. IGF-1 can promote osteoclast differentiation through maintaining the normal interplay between osteoblasts and osteoclast precursors, depending on regulating the production of RANKL and M-CSF (Wang et al., 2006). Furthermore, IGF/IGF-IR signaling mediates the proliferation and differentiation of osteoprogenitor regulated by PTH, influencing periosteal bone formation (Wang et al., 2007).

Mounting evidences have suggested that gut microbiota affects host development and general health *via* regulating systemic IGF-1 levels (Yan and Charles, 2018). Commensal microbe *Acetobacter pomorum* regulates insulin/IGF signaling in Drosophila depending on the activity of pyrroloquinoline quinone-dependent alcohol dehydrogenase, contributing to host development and metabolic homeostasis (Shin et al., 2011). During intestinal or pneumonic infection, gut bacterium *Escherichia coli* O21:H^+^ maintains systemic IGF-1 level, which links to increased IGF-1 level and *E.coli* O21:H^+^ colonization in white adipose tissue, thus preventing muscle wasting and promoting disease tolerance (Schieber et al., 2015). The catabolic and anabolic effects of commensal gut microbiota on bone remolding are also partially mediated by regulation of systemic and local IGF-1 signaling (Yan et al., 2016; Novince et al., 2017). Gut microbiota colonization in GF mice promotes bone turnover with elevated IGF-1 levels in serum and bone marrow, while antibiotic treatment in SPF mice reduces serum IGF-1 level and increases bone mass, which can be rescued by SCFA supplement (Yan et al., 2016).

#### Sex Hormones

Sex hormones, such as follicle-stimulating hormone (FSH), estrogen, and androgen, have been considered as important regulators of bone metabolism (Leder, 2007; Khosla, 2020). Both estrogen and androgen prevent bone resorption and sustain bone formation (Syed and Khosla, 2005). A clinical randomized study demonstrated that hormone/estrogen replacement therapy could ameliorate postcranial bone density and alveolar bone mass in postmenopausal women (Civitelli et al., 2002). Furthermore, estrogen deficient rats had lower periodontal bone density than control rats, while estradiol treatment protects against alveolar bone loss induced by estrogen deficiency (Duarte et al., 2006). Additionally, estrogen deficiency has adverse effects on the bone healing after bone extraction (Bezerra et al., 2013). Estrogen doesn’t directly regulate osteoclast differentiation, instead it inhibits mature osteoclast function by downregulating genes relevant to bone resorption, and controls mature osteoclast lifespan by promoting apoptosis in binding with estrogen receptor α (ERα) (Imai et al., 2009). Consistently, estrogen-deficient rats presented increased osteoclasts in alveolar bone, and exogenous estrogen supplement reduces alveolar bone osteoclasts partially due to osteoclast apoptosis (Faloni et al., 2007; Bezerra et al., 2013). Of note, instead of femur and vertebrae, estrogen affects maxillary bone phenotype *via* IL-33/ST2 signaling in a site-specific manner (Macari et al., 2018). In addition, without direct action on bone cells, high FSH activity promotes bone formation depending on ovary pathway (Allan et al., 2010).

Consistently, gut microbiota has been suggested as an important regulator of hormone homeostasis (Baker et al., 2017; Rizzetto et al., 2018). As early as about 40 years ago, it was reported that gut microbiota intervention by antibiotic treatment resulted in increased fecal estrogen levels with reduced urinary estrogen excretion, especially estradiol and estriol, which potentially related to the suppressed gut reductive estrogen metabolism (Adlercreutz et al., 1984). More evidences have shown the association between gut microbial composition and urinary or systemic levels of estrogen and estrogen metabolites (Flores et al., 2012; Fuhrman et al., 2014). As for testosterone, early-life colonization of gut commensal microbes or gut microbial transfer alters serum level of testosterone, affecting the development of autoimmune diseases (Markle et al., 2013). However, the mechanism whereby gut microbiota affects hormone remains uncertain. Recently, it has been reported that gut microbiota affects hormone homeostasis depending on “endobolome,” a novel term that refers to the aggregate of gut microbial genes involved in the production and metabolism of sex steroid hormones including estrogen (Aguilera et al., 2020). Gut microbiota metabolizes hormones *via* distinct enzymes, such as β-glucuronidases, β-glucuronides, and hydroxysteroid dehydrogenase, affecting circulating and local hormone levels (Plottel and Blaser, 2011; García-Gómez et al., 2013). Enzyme β-glucuronidases derived from gut microbiota promote deconjugated estrogens production, which travel into circulation and carry out physiological functions (Rizzetto et al., 2018). Additionally, gut microbe *Clostridium scindens* could transfer glucocorticoids to androgens *via* side-chain cleavage (Ridlon et al., 2013). Some gut bacteria, mostly belonging to the family *Coriobacteriaceae*, are capable of metabolizing soy-derived isoflavone-glycosides and producing metabolites, including equol, a compound with strong estrogenic activity (Guadamuro et al., 2017; Chen and Chen, 2021). Although direct evidence is still lacking, interplays in “gut-hormone-bone” axis provide another pathway whereby gut microbiota regulates periodontal diseases.

#### Serotonin

Serotonin, namely, 5-hydroxytrypatamine (5-HT), is a neurotransmitter with hormone-like activity, which can affect bone homeostasis *via* circulation. Circulating serotonin derived from duodenum inhibits bone formation and regulates bone mass (Yadav et al., 2008). Serotonin transporter is a plasma membrane transporter expressing on bone cells, which is responsible for the uptake of extracellular serotonin and subsequent intracellular serotonin storage and degradation. Extracellular serotonin signaling enhancement *via* serotonin transporter inhibition exerts detrimental effects on skeletal growth and bone homeostasis, as indicated by reduced bone mass, altered microarchitecture, as well as impaired mechanical properties, relating to increased risk of osteoporotic fracture (Warden et al., 2005; Warden et al., 2008; Eom et al., 2012; Wu et al., 2012; Kerbage et al., 2014; Gorgas et al., 2021). Serotonin can promote osteoclast proliferation and differentiation *via* NF-κB signaling and influence osteoblast proliferation in a dose-dependent manner (Battaglino et al., 2004; Gustafsson et al., 2006). Serotonin directly prevents osteoblast differentiation *via* Htr1b and CREB signaling (Yadav et al., 2008).

Serotonin is mainly synthesized by enterochromaffin cells and exerts biological effects *via* the circulation. Accumulating evidences indicate that gut microbiota affects the biosynthesis and availability of serotonin. SCFAs derived from gut microbiota, such as acetate and butyrate, promote serotonin secretion *via* upregulating the expression of tryptophan hydroxylase 1 (Tph1), which is the rate-limiting enzyme responsible for serotonin biosynthesis in enterochromaffin cells (Reigstad et al., 2015; Vincent et al., 2018). Gut microbe *Clostridium ramosum* or its components are also reported to increase serotonin secretion and availability from enterochromaffin cells (Mandić et al., 2019). Hence, gut microbiota also potentially regulates alveolar bone homeostasis *via* serotonin.

## Conclusion

Periodontitis is characterized by inflammatory periodontal bone loss resulting from impaired alveolar bone homeostasis, which is influenced by multiple factors including systemic diseases or local intestinal infections. The deleterious effects of systemic or intestinal diseases on alveolar bone induce complexity of periodontitis treatment and inferior prognosis. Recent findings have revealed the pivotal roles of gut microbiota in bone homeostasis and pathologies, providing a potential mechanism whereby systemic conditions affect periodontitis. Given the crosstalk between gut microbiota and host, alteration in gut microbiota due to systemic disorders may regulate alveolar bone homeostasis. Future investigations are needed to provide adequate evidences and advance the understanding of “gut-alveolar bone” axis, thus promoting the better management of periodontitis.

## Author Contributions

XX contributed to the conception and manuscript revision, read, and approved the submitted version. XJ wrote the first draft of the manuscript and contributed to manuscript revision, read, and approved the submitted version. Other authors contributed to manuscript revision, read, and approved the submitted version.

## Funding

This work was supported by National Natural Science Foundation of China (81870754, 81800989, 81991500, 81991501), and a research grant from the West China Hospital of Stomatology, Sichuan University (LCYJ2019-4).

## Conflict of Interest

The authors declare that the research was conducted in the absence of any commercial or financial relationships that could be construed as a potential conflict of interest.

## Publisher’s Note

All claims expressed in this article are solely those of the authors and do not necessarily represent those of their affiliated organizations, or those of the publisher, the editors and the reviewers. Any product that may be evaluated in this article, or claim that may be made by its manufacturer, is not guaranteed or endorsed by the publisher.
